# Analysis of ancient and modern horse genomes reveals the critical impact of lncRNA-mediated epigenetic regulation on horse domestication

**DOI:** 10.3389/fgene.2022.944933

**Published:** 2022-10-05

**Authors:** Baoyan Xu, Guixian Yang, Baowei Jiao, Hao Zhu

**Affiliations:** ^1^ Bioinformatics Section, School of Basic Medical Sciences, Southern Medical University, Guangzhou, China; ^2^ Medical Engineering Department, Integrated Hospital of Traditional Chinese Medicine, Southern Medical University, Guangzhou, China; ^3^ Zhujiang Hospital, Southern Medical University, Guangzhou, China; ^4^ State Key Laboratory of Genetic Resources and Evolution, Kunming Institute of Zoology, Chinese Academy of Science, Kunming, China

**Keywords:** domestication, lncRNA, epigenetic regulation, neural crest cell, horse

## Abstract

**Background:** The domestication of horses has played critical roles in human civilizations. The excavation of ancient horse DNA provides crucial data for studying horse domestication. Studies of horse domestication can shed light on the general mechanisms of animal domestication.

**Objective:** We wish to explore the gene transcription regulation by long noncoding RNAs (lncRNAs) that influence horse domestication.

**Methods:** First, we assembled the ancient DNA sequences of multiple horses at different times and the genomes of horses, donkeys, and Przewalski horses. Second, we extracted sequences of lncRNA genes shared in ancient horses and sequences of lncRNA genes and the promoter regions of domestication-critical genes shared in modern horses, modern donkeys, and Przewalski horses to form two sample groups. Third, we used the LongTarget program to predict potential regulatory interactions between these lncRNAs and these domestication-critical genes and analyzed the differences between the regulation in ancient/modern horses and between horses/donkeys/Przewalski horses. Fourth, we performed functional enrichment analyses of genes that exhibit differences in epigenetic regulation.

**Results:** First, genes associated with neural crest development and domestication syndrome are important targets of lncRNAs. Second, compared with undomesticated Przewalski horses, more lncRNAs participate in the epigenetic regulation in modern horses and donkeys, suggesting that domestication is linked to more epigenetic regulatory changes. Third, lncRNAs’ potential target genes in modern horses are mainly involved in two functional areas: 1) the nervous system, behavior, and cognition, and 2) muscle, body size, cardiac function, and metabolism.

**Conclusion:** Domestication is linked to substantial epigenetic regulatory changes. Genes associated with neural crest development and domestication syndrome underwent noticeable lncRNA-mediated epigenetic regulation changes during horse domestication.

## 1 Introduction

The domestication of animals and plants has greatly changed human life. For example, pigs and cattle provide food for humans, cats and dogs are companions for people, wheat and rice are the main food crops for humans (Kilian et al., 2010), and horses and camels are important means of human transportation (Vigne, 2011; Zeder, 2012). Researchers study animal domestication mainly using two methods. One method is based on archaeological records, which analyze the changes in animal body structure based on fossils from different periods and locations (Conolly et al., 2011). Animal skeletal changes, age at death, sex ratio, and isotopic characteristics can be used as indicators for analyzing domestication in archaeological studies. However, archaeological data are fragmented, and many characteristic differences may have already existed between domesticated animals and their wild ancestors in the early domestication phase, including coat color, temperament, and reproductive ability, which cannot be discovered through skeletal archaeology. The other method analyzes the genome of modern domesticated animals to obtain temporal and geographic information on domestication and to discover the genetic basis of domestication (Luikart et al., 2001; Vilà et al., 2001; Pedrosa et al., 2005; Naderi et al., 2008), including inferring the initial domestication centers based on the genetic diversity generated by geographical differences. A large amount of phenotypic diversity exists among and within species of modern domesticated animals, and genomic analysis can reveal the influence of specific loci on phenotypes, including coat color, body size, fat content, biological clock, and behavior (Wright, 2015). However, genomic information obtained from living animals can reveal only a small fraction of domestication information. Over the past decade, researchers have gained access to a wealth of genomic information from ancient species using new sequencing techniques. The first extraction of ancient animal DNA, an extinct zebra species, was achieved in the 1980s (Higuchi et al., 1984). Since then, the combined archaeological and genomic analysis have led to a better understanding of the role of location, timing, and artificial selection in the domestication of animals.

Domesticated mammals share common traits. Especially, in the early stage, all domesticated mammals yield reduced fear and reactive aggression. Wilkins et al. (2014) focused on a special group of cells found in embryos, neural crest cells (NCCs), proposed that the genetic changes affecting their development were at the root of vertebrate domestication, and termed these traits the “neural crest/domestication syndrome” (NCDS). NCC-derived tissues include the major parts of the jaws and teeth, pigmentation cells, components of the external ears, and cells involved in sympathetic responses docility. Many genes are required for NCC formation and development (which are referred to as neural crest/domestication syndrome-associated genes, or NCDS genes in this study), but their roles in the NCC genetic regulatory network remain poorly known (Simoes-Costa and Bronner, 2015). It is proposed that the neural crest cell contribution to final target tissues could be modified by mutations (Wilkins et al., 2021), but it is questionable whether sufficient mutations can be generated and accumulated in a short period for domestication. Whether the NCDS hypothesis provides a unified explanation for domestication, these genes should be important targets of combined archaeological and genomic analysis for revealing the mechanisms of animal domestication.

The domestication of horses not only revolutionized the way humans migrated but also changed the way human activities and diseases were spread and goods were distributed, allowing for the first real globalization of humankind (Kelekna, 2009). Fossils of horses at all stages of domestication have been excavated and reported (Orlando, 2015), with related studies reporting the time and place of horse domestication (Orlando, 2020; Librado et al., 2021). Early studies of ancient DNA analysis focused on protein-coding genes, and no computational studies on lncRNAs and their potential epigenetic target genes have been reported. However, most protein-coding genes are highly conserved in mammals and would unlikely have changed much in just a few thousands of years. We posit that it should be the regulation of the expression of these genes, but not the sequence of these genes, that undergoes significant changes during domestication. Gene expression is regulated by multiple mechanisms, including transcriptionally by transcription factors and long noncoding RNAs (lncRNAs). Many lncRNAs can bind to DNA sequences by forming RNA: DNA triplexes and recruit epigenomic modification enzymes to DNA binding sites. Thus, transcription factors and their DNA-binding sites and lncRNAs and their DNA-binding sites are two mechanisms controlling gene expression changes. Mammalian lncRNA genes are less conserved, and many are species-specific (Ulitsky, 2016; Sarropoulos et al., 2019), suggesting that they may have a greater effect on gene expression than transcription factors within a short period. Therefore, they should be essential regulators of domestication. An example from dog domestication suggests that diverse phenotypic changes can be produced in a short time without much change in the genome (Larson et al., 2012; Botigué et al., 2017; Pendleton et al., 2018)

In this study, we used two groups of published data to analyze the effects of lncRNAs on horse domestication. One group contains the DNA sequences from ancient and modern horses, and the other group contains the DNA sequences of modern horses, modern donkeys (a closely related species to horses), and Przewalski horses. The Przewalski horse was once assumed to be the only surviving wild horse (Schubert et al., 2014b), but the sequencing of its genome and subsequent genome analysis in 2018 revealed that it is actually a descendant of the ancient Botai horse. In other words, the Przewalski horses are semi-domesticated or undomesticated horses (Gaunitz et al., 2018); thus, it provides important information on horse domestication when combined with ancient horse DNA. The promoter regions of genes are the most important binding sites of lncRNAs and are critical for gene expression. To make data analyses less influenced by ancient DNA’s relatively poor sequencing quality, we only used promoter regions to predict lncRNAs’ binding sites and analyze lncRNA-mediated gene expression regulation. Our results support the hypothesis that lncRNAs and NCDS genes significantly influenced the domestication of horses. The results also indicate that domestication is associated with an increase in epigenetic regulation.

## 2 Methods

### 2.1 Samples and candidate genes

**FIGURE 1:**
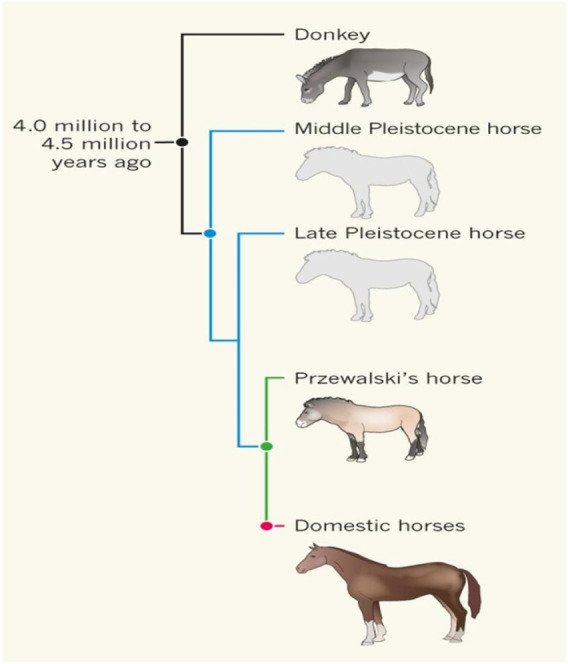
The relationships between ancient horses, Przewalski horses, modern horses, and donkeys [from (Miller and Lambert, 2013) with permission]. Przewalski horses are undomesticated descendent of the ancient horse Botai_2_5500.

We examined the DNA sequencing data of 8 ancient horses (Table 1). Ancient DNA sequencing results with relatively good quality were obtained from three ancient horses in three typical periods: a 40,000-year-old wild horse (CGG10022), a 5,500-year-old Botai horse (Botai_2_5500), and a 2300-year-old ancient horse (BER09_I). CGG10022 was undomesticated, and BER09_I was largely (but not completely) domesticated. These ancient genomes, together with the genome of modern horses, form a chronological data group (called the “CGG/Botai/BER/modern horse group”). Moreover, the genomes of modern horses, modern donkeys, and Przewalski horses form an interspecific data group (called the “modern horse/modern donkey/Przewalski horse group”) (Table 1; Figure 1).

**TABLE 1:** Sample information.

Sample	Age (BP or BCE)	Genome/GFF version	Assembly level	Source
CGG10022	42,012 (BCE)	—	Shotgun reads	Schubert et al. (2014b)
Botai_2_5500	5,500	—	BAM(2.0)	Gaunitz et al. (2018)
Borly4_PAVH8_4978	4,071	—	BAM(2.0)	Gaunitz et al. (2018)
838-64_NB46	4,978	—	Shotgun reads	Librado et al. (2017)
UushgiinUvur_Mon84_312	3,123	—	BAM (2.0)	Gaunitz et al. (2018)
BER09_I	2,300	—	Shotgun reads	Librado et al. (2017)
I-K3_Arz2	2,760	—	Shotgun reads	Librado et al. (2017)
Yenikapi_Tur172_1695	1,695	—	BAM (2.0)	Gaunitz et al. (2018)
Modern horse		EquCab3.0	Genome	NCBI
Modern donkey		ASM303372v1	Scaffold	NCBI
Przewalskii horse		GCA_000696695.1	Scaffold	NCBI

Data download links or accession numbers: CGG10022: https://www.ebi.ac.uk/ena/browser/view/PRJEB7537; Botai_2_5500: https://www.ebi.ac.uk/ena/browser/view/PRJEB22390; BER09_I: https://www.ebi.ac.uk/ena/browser/view/PRJEB19970; Modern horses: https://www.ncbi.nlm.nih.gov/assembly/GCF_002863925.1/; Modern donkey: https://www.ncbi.nlm.nih.gov/assembly/GCA_003033725.1/; Przewalskii horse: https://www.ncbi.nlm.nih.gov/assembly/GCF_000696695.1/

Researchers identified 125 genes with positive selection signals related to horse domestication (Supplementary Table S1) (Schubert et al., 2014b). These genes can be divided into two groups. One group of genes is involved in muscle and limb development, joint connectivity, and the cardiac system; the other is involved in cognitive functions, including social behavior, learning ability, fear response, and conformation. These genes are considered essential common genes involved in domestication. Librado et al. (2017) found that the NCDS genes (Supplementary Table S2), 15 of which have been reported in the relevant literature (Wilkins et al., 2014), underlie the phenotypic differences between wild and domesticated horses. We examined the intersection of the 125 positively selected genes (Schubert et al., 2014b) in the modern horse/modern donkey/Przewalski horse group and the intersection of the 15 NCDS genes (Wilkins et al., 2014) in the modern horse/modern donkey/Przewalski horse group, respectively. 73 out of the 125 positive selection-related genes and 10 out of the 15 NCDS genes were identified in all three species (Supplementary Table S3).

### 2.2 Extracting ancient DNA sequences of long noncoding RNAs and promoter regions of genes

**FIGURE 2:**
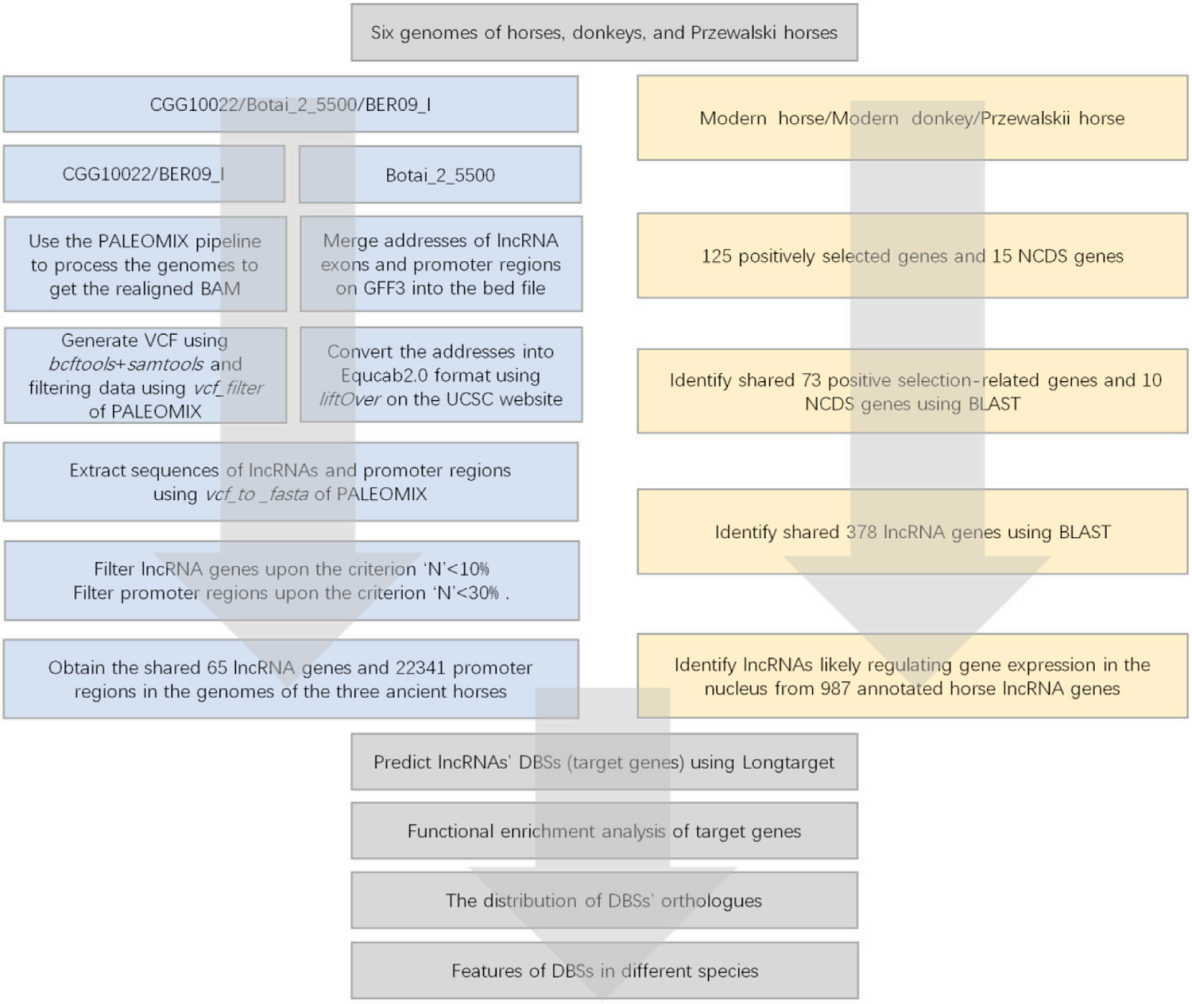
The flowchart of data processing. The left panel indicates the data processing of the CGG/Botai/BER/modern horses group; the right panel indicates the data processing of the modern horses/donkeys/ Przewalski horses group.

Since promoter regions are the most important regions for lncRNA-mediated epigenetic regulation, we only analyzed the promoter regions of candidate genes. In the CGG/Botai/BER/Modern horse group, the DNA sequencing results of CGG10022 and BER09_I were shotgun reads, so we processed all data with the following steps (Figure 2). 1) All data of ancient horse genomes were processed using the ancient DNA sequence processing pipeline PALEOMIX (Version 1.2.13.2) (Schubert et al., 2014a). 2) Then, lncRNA genes and promoter regions of candidate genes were identified. 3) Ancient DNA reads were aligned to the modern horse reference genome EquCab3.0 to generate VCF files. 4) The location of each gene’s transcription start site (TSS) was determined according to the EquCab3.0 gene annotation file. 5) The FASTA sequence of the ancient DNA was generated using the *vcf_to_fasta* command of PALEOMIX. 6) Sequences −3,500 bp–+1500 bp upstream/downstream of the TSS of the candidate genes were obtained as the promoter regions. 7) The exon sequences of each lncRNA were obtained and assembled into lncRNA sequences.

The DNA sequencing results of Botai_2_5500 have a pairwise sequence alignment with the modern horse genome EquCab2.0. However, no lncRNA genes are annotated in EquCab2.0. We used the *liftover* tool in the UCSC Genome Browser (http://genome.UCSC.edu) to convert the positions of exons in EquCab3.0 to those in EquCab2.0 and then used PALEOMIX’s *vcf_to_fasta* to generate the FASTA sequences and obtain the lncRNA sequences. The promoter regions of the candidate genes were similarly obtained, which ranged from −3,500 bp–+1500 bp upstream/downstream of the TSS.

In the modern horse/modern donkey/Przewalski horse group, promoter regions (−3,500 bp–+1500 bp upstream/downstream of the TSS) and lncRNA sequences were extracted based on genome positions in the downloaded genome sequence files in FASTA format and gene annotation files.

### 2.3 Filtering of the long noncoding RNA genes and promoter regions of candidate genes

The DNA sequences of CGG/Botai/BER ancient horses have considerable “N”s. We chose only lncRNA genes with “N” < 10% to filter out unreliable lncRNA genes. Upon this criterion, we identified 65 lncRNA genes in the intersection between CGG10022, Botai_2_5500, BER09_I, and modern horses (Supplementary Table S4). Upon the criterion “N” < 30%, we identified 22,341 promoter regions in the three ancient horses (CGG/Botai/BER ancient horses).

The genomes of the modern horse/donkey/Przewalski horse contain many lncRNA genes, but what lncRNAs function in the nucleus and in the cytoplasm are unknown. A lncRNA capable of regulating gene expression in the nucleus may bind to duplex DNA sequences by forming triplexes and recruit epigenomic modification enzymes to binding sites. Each triplex comprises triplex-forming oligonucleotides (TFO) in the lncRNA and a triplex-targeting site (TTS) in the DNA sequence. Overlapping TFOs indicate a DNA binding domain (DBD) in the lncRNA, and overlapping TTSs indicate a DNA binding site (DBS) in the DNA sequence. We first used the Triplexator program (which is very fast) to predict lncRNAs’ TTSs in promoter regions genome-wide (Buske et al., 2012). We assumed that if a lncRNA had > 2,000 TTSs, the lncRNA likely regulate gene expression in the nucleus. This empirical cutoff helped us choose the proper lncRNAs. A total of 4,562 lncRNA genes were annotated in the horse genome, 987 of which had > 2,000 TTSs. In addition, if multiple DBDs were predicted in a lncRNA, only the DBSs that correspond to the best DBDs (which have the most DBSs) were analyzed. Compared with the horse genome, only 900 lncRNA genes were annotated in the donkey genome. Due to the lack of annotated lncRNA genes in donkeys and Przewalski horses, we used the BLAST program to search the 987 horse lncRNAs against the modern donkey and Przewalski horse genomes (Johnson et al., 2008). The search identified 378 homologous lncRNA genes in modern horses, modern donkeys, and Przewalski horses (Supplementary Table S5).

### 2.4 Predicting DBSs in the promoter regions of genes

In all, 65 lncRNA genes and 22,341 promoter regions were obtained from the genomes of the CGG/Botai/BER/Modern horse group, and 378 lncRNA genes and the promoter regions in the 83 key domestication-related genes were obtained from the genomes of the modern horse/modern donkey/Przewalski horse group. We used the LongTarget program we developed previously (Lin et al., 2019) to predict the DBSs of these lncRNAs in these promoter regions, which helped us find which genes might be the targets of these lncRNAs. In total, 4 (samples) × 65 (lncRNAs) × 22,341 (promoter regions) (Supplementary Material S1–S4) and 3 (samples) × 378 (lncRNAs) × 83 (promoter regions) predictions were performed (Supplementary Material S5–S7). The default LongTarget parameters were used (*Identity* ≥ 60%, which means > 60% RNA: DNA bases are paired, and *Minimal triplex* ≥ 50, which means DBSs ≥ 50 bp).

We computed the binding affinity for each pair of lncRNA and DBS to measure the strength of lncRNA binding. Binding affinity was defined as the product of DBS length and the averaged Identity values of all triplexes at the site. Thus, a binding affinity ≥ 60 means DBS ≥ 100 bp if the Identity = 60%. In the modern horse/modern donkey/Przewalski horse group, only DBSs with affinity > 60 were analyzed.

To check whether the sequencing quality of ancient horses influences DBS prediction, we examined DBSs’ mean length in the three ancient horses and in the three modern species. The results indicate that DBSs have comparable lengths in the two groups (Table 2).

**TABLE 2:** LncRNAs’ number and DBSs’ number, mean length, and mean affinity value in samples in the two groups.

Species	LncRNA number	DBS number	Mean DBS length	Mean binding affinity
CGG10022	65	70,445	140	89
Botai_2_5500	65	49,598	120	77
BER09_I	65	54,283	130	82
Modern horse	376	28,825	157	101
Modern donkey	376	22,537	156	100
Przewalski horses	319	11,007	136	87

DBSs, in ancient horses were predicted in 22,341 promoter regions, and DBSs, in modern horses, modern donkeys, and Przewalski horses were predicted in the promoter regions of 378 genes.

### 2.5 Calculating sequence differences in the long noncodingRNAs in the CGG/Botai/BER/Modern horse group

To estimate lncRNAs’ sequence differences in the CGG/Botai/BER/Modern horse group, we first performed multiple sequence alignment using Clustalw2 (Larkin et al., 2007). Then, we used the Phylip program (http://evolution.genetics.washington.edu/phylip.html) to calculate the sequence differences.

### 2.6 Identification of the species specificity of DBSs

To examine whether lncRNAs’ target genes are conserved, we used each DBS in the modern horse genome as the query sequence to search for orthologous sequences in the genomes of modern donkeys and Przewalski horses. The sequence search was performed using the BLAST program, and the 100,000 bp region upstream/downstream of the TSS of the corresponding gene was the target region. If no orthologous sequence was obtained for a DBS, the DBS was assumed to be modern horse specific.

### 2.7 Analysis of the functional enrichment of target genes

The functions of genes in horses and donkeys are poorly annotated because published studies remain very limited. We determined the functions of horse and donkey protein-coding genes based on the GeneCards Human Gene database (https://www.genecards.org/), assuming that these protein-coding genes have the same functions in humans, horses, and donkeys.

We performed functional enrichment analysis for the epigenetic target genes of lncRNAs in both the CGG/Botai/BER/Modern horse group and the modern horse/modern donkey/Przewalski horse group using the g: Profiler program (https://biit.cs.ut.ee/gprofiler/gost) (Raudvere et al., 2019). For the modern horse/modern donkey/Przewalski horse group, functional enrichment analysis was applied to the genes shared by the three species and the genes specific to modern horses, respectively (g:Profiler parameters: Organism = Equus caballus, Ordered query = No, Statistical domain scope = All known genes, Statistical threshold = Benjamini–Hochberg FDR, User threshold = 0.05, 2< term size < 2,000).

## 3 Results

### 3.1 Domestication is associated with an increase in epigenetic regulation

We used the genome data of the three ancient horses to analyze the regulatory function of lncRNAs on gene expression in ancient horses and used the genome data of modern horses, modern donkeys, and Przewalski horses to analyze the regulatory function of lncRNAs in modern horses and related species (Table 1). Given that most protein-coding genes have unlikely evolved much in the short domestication period, we postulated that gene expression linked to horse domestication has been more subject to epigenetic regulation. In the CGG/Botai/BER/Modern horse group, 65 lncRNA genes and 22,341 promoter regions of genes were analyzed. We first examined whether lncRNA sequences show clear divergence across ancient and modern horses. Agreeing with that predicted DBSs in ancient and modern horses have a comparable length (Table 2), the computed sequence differences indicated that these lncRNA sequences have limited variation (especially in ancient horses) (Table 3), from which we infer that it is their DBSs that may have played important roles in the changes of domestication-related epigenetic regulation.

**TABLE 3:** Distances between the 65 lncRNAs in the CGG/Botai/BER ancient horse group.

Sample	CGG10022	Botai_2_5500	BER09_I	Modern horse
CGG10022	0	0.000285	0.000417	0.000926
Botai_2_5500	0.000285	0	0.000288	0.000547
BER09_I	0.000417	0.000288	0	0.000561
Modern horse	0.000926	0.000547	0.000561	0

**FIGURE 3:**
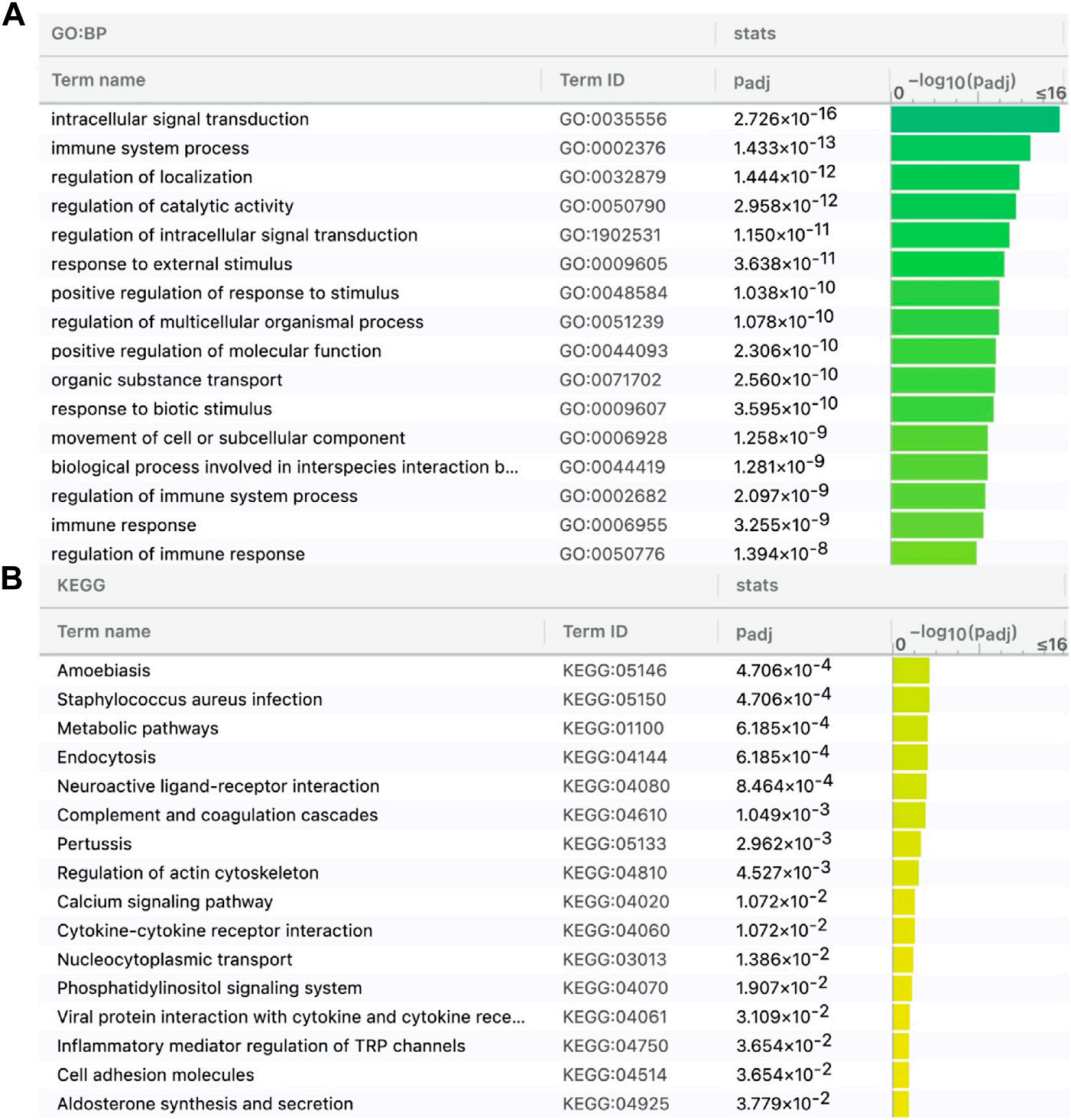
Target genes of the 65 lncRNAs in the CGG/Botai/BER/modern horses are enriched in immune, metabolism, and neuroactive ligand-receptor interaction related GO terms **(A)** and KEGG pathways **(B)**.

Then, we predicted lncRNAs’ DBSs, assuming that genes at DBSs were lncRNAs’ target genes. For the CGG/Botai/BER/Modern horse group, 65 lncRNAs had DBSs in the promoter regions of 2,988 genes (Supplementary Table S6). For example, the lncRNA LOC106783351 had 1104 DBSs in 644 protein-coding genes and 358 noncoding genes, and the lncRNA LOC11773204 had 1492 DBSs in 657 protein-coding genes and 327 noncoding genes. The target genes with high DBS binding affinity were mainly immune and nervous system-related. The functional enrichment analysis indicates that target genes are mainly enriched in immune, metabolism, and response to external stimulus-related GO terms (Figure 3A) and metabolism and neuroactive ligand-receptor interaction pathways (Figure 3B). Notably, more DBSs were predicted in the undomesticated CGG10022 (than in the semi-domesticated Botai_2_5500 and BER09_I), but in modern species, more DBSs were predicted in the domesticated horses (Table 2). This is confirmed when we examined DBSs in the NCDS genes. This result could indicate a substantial turnover of DBSs during domestication.

Two results were obtained from the modern horse/donkey/Przewalski horse group. First, of the 378 lncRNAs shared in the three species, 376 in modern horses and donkeys but 319 in Przewalski horses have DBSs, indicating that more lncRNAs have functions in the domesticated horse and donkey. The 376 lncRNAs have 28,825 and 22,537 DBSs in 83 positively selected or NCDS genes in modern horses and modern donkeys, but the 319 lncRNAs have only 11,007 DBSs in the 83 genes in Przewalski horses (Tables 2, 4). Second, to confirm that domesticated horses acquired specific epigenetic regulation, we used the BLAST program to perform six pairwise sequence searches to examine how many DBSs obtained in a species are also obtained in the other two species. Since DBS sequences are quite long, these BLAST searches basically yielded unique high-scoring hits. The results indicate that modern horses share many DBSs with donkeys but not with Przewalski horses (Table 4). That is, many DBSs in modern horses and donkeys do not have homologous sequences in Przewalski horses. However, most DBSs in Przewalski horses have homologous sequences in modern horses and donkeys. These results indicate an increase in epigenetic regulatory relationships in modern horses and donkeys and suggest that the increased regulatory relationships may have greatly influenced the domestication of horses and donkeys.

**TABLE 4:** Orthologues of DBSs in different species.

Pairwise BLAST search of DBSs	Hits	No hits
28,825 DBSs in horses → Przewalski horses	15,069 (52.2%)	13,756 (47.7%)
28,825 DBSs in horses → donkeys	21,044 (73.0%)	7,781 (27.0%
22,536 DBSs in donkeys → Przewalski horses	13,990 (62.1%)	8,546 (37.9%)
22,536 DBSs in donkeys → horses	18,588 (82.5%)	3,949 (17.5%)
11,007 DBSs in Przewalski horses → donkeys	9,994 (90.8%)	1,013 (9.2%)
11,007 DBSs in Przewalski horses → horses	9,416 (85.6%)	1,591 (14.5%)

### 3.2 Epigenetic regulation is stronger in domesticated horses

**FIGURE 4:**
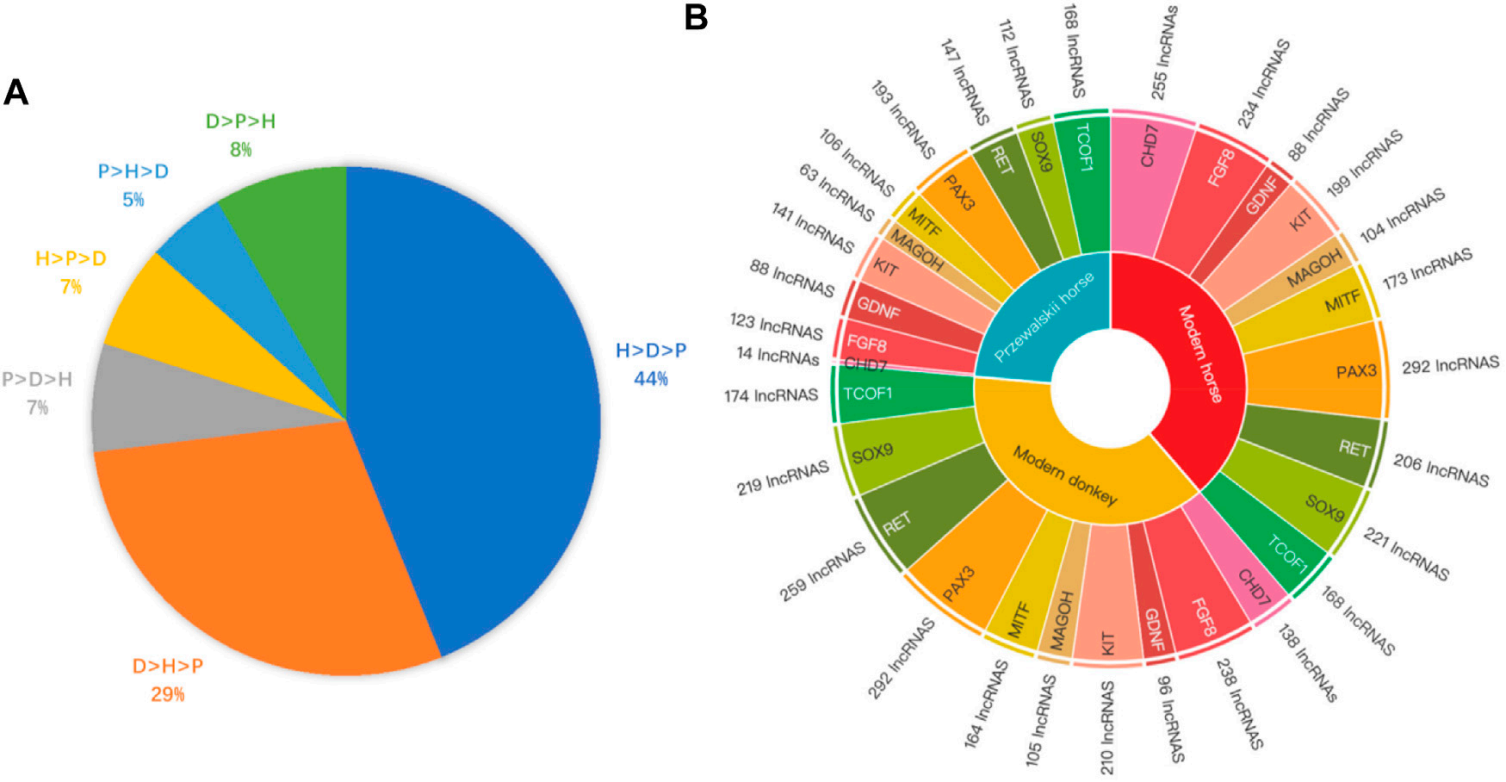
The Binding affinity of DBSs in species and the number of DBSs in different NCDS genes. **(A)** LncRNAs’ mean binding affinity at DBSs in modern horses, modern donkeys, and Przewalski horses (indicated by H, D, and P, respectively). The label “H > D > P 44%” indicates that the mean binding affinity of 44% of the lncRNAs has the largest values in modern horses but the smallest values in Przewalski horses. Similarly, the label “D > H > P 29%” indicates that the mean binding affinity of 29% of the lncRNAs has the largest values in modern donkeys but the smallest values in Przewalski horses. **(B)** The regulatory relationship between the 319 lncRNAs and the 10 NCDS genes in modern horses, modern donkeys, and Przewalski horses. Species in the inner layer correspond to the 10 NCDS genes in the second layer, and NCDS genes in the second layer correspond to lncRNA numbers in the outer layer.

**FIGURE 5:**
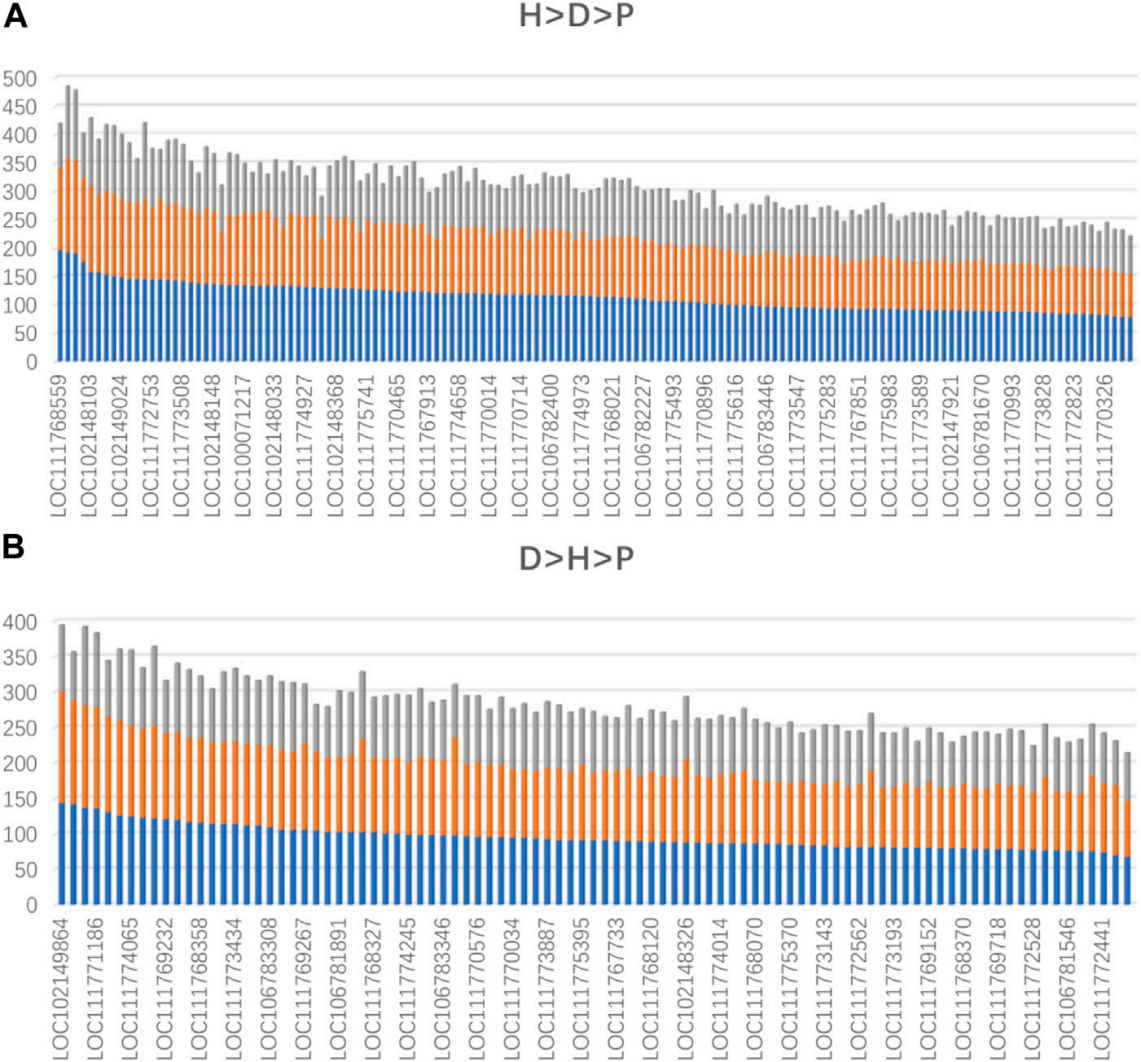
The lncRNAs whose average binding affinity shows a consistent pattern in modern horses, modern donkeys, and Przewalski horses (indicated by blue, orange, and gray colors, respectively). Values on the vertical axis indicate binding affinity values. The names of some lncRNAs are indicated (Supplementary Table S7, S8). **(A)** The lncRNAs whose average binding affinity shows the H > D > P pattern. **(B)** The lncRNAs whose average binding affinity shows the D > H > P pattern.

To further confirm that domestication is associated with epigenetic regulation, we analyzed the binding affinity of the DBSs in the modern horses/modern donkeys/Przewalski horses group. Excluding lncRNAs with very few DBSs, we calculated the average binding affinity of all DBSs for the 319 lncRNAs. In all, 140 lncRNAs (44%) showed the binding affinity pattern of “modern horse > modern donkey > Przewalski horse”, and 93 lncRNAs (29%) showed the binding affinity pattern of “modern donkeys > modern horses > Przewalski horses” (Supplementary Tables S7, S8) (Figure 4A). This result implies that a majority of the lncRNAs show stronger epigenetic regulation in domesticated animals (horses and donkeys) than in undomesticated animals (Przewalski horses). When examining detailed lncRNAs, it is clear that the average binding affinity of lncRNAs in horses and donkeys was higher than that in Przewalski horses (Figure 5).

### 3.3 Regulation of genes associated with neural crest/domestication syndrome

In the modern horse/modern donkey/Przewalski horse group, we further analyzed the potential regulatory relationships between the 10 NCDS genes and the 376 lncRNAs. Of note, each NCDS gene (except CHD7) has DBSs of > 60 lncRNAs, and more lncRNAs have DBSs in NCDS genes in modern horses and modern donkeys than in Przewalski horses (the total DBSs of lncRNAs in the 10 genes are 1,940, 1,895, and 1,155 in the modern horses, donkeys, and Przewalski horses) (Figure 4B). Also, 9 out of the 10 NCDS genes have fewer regulatory lncRNAs in Przewalski horses than in modern horses and modern donkeys. These results indicate that the increased epigenetic regulation in domesticated animals is enriched at least in NCDS genes, and this enrichment lends a support for the “neural crest/domestication syndrome” hypothesis.

The NCDS genes *FGF8*, *KIT*, *PAX3*, *RET,* and *SOX9* were predicted to be regulated by > 50% of the lncRNAs in both modern horses and donkeys. *FGF8* is related to the cardiovascular system and craniofacial phenotypes. *KIT* is related to pigmentation and integument phenotypes. *PAX3* is related to pigmentation and nervous system phenotypes. *RET* is related to renal/urinary system and digestive/alimentary phenotypes. *SOX9* is related to skeleton, growth/size/body region and limb/digit/tail phenotypes. The intensive regulations of these genes (some of which may have been acquired recently) may help explain the following phenotypic changes: 1) changes in coat color (Orlando, 2020): in ancient times, a spotted coat was very common, but by the Middle Ages it was replaced by a uniform chestnut coat color from red to brown; 2) rapid dental evolution (MacFadden and Ceding, 1994): as the food source changed, shifting from young leaves to herbs, the teeth became more worn, and in turn, the crowns were raised, and the teeth became larger; 3) changes in the craniofacial area: as the teeth changed, the upper and lower jaws of the teeth also became larger and longer, creating the typical long face; and 4) changes in the toes: this was an important change in the evolutionary process of the horse (Solounias et al., 2018); eohippus had three toes on the hind limbs and four toes on the forelimbs, while the modern horse now walks with a single toe, which greatly enhances its running ability.

### 3.4 Modern horses have undergone specific evolution of epigenetic regulation in the nervous system

**FIGURE 6:**
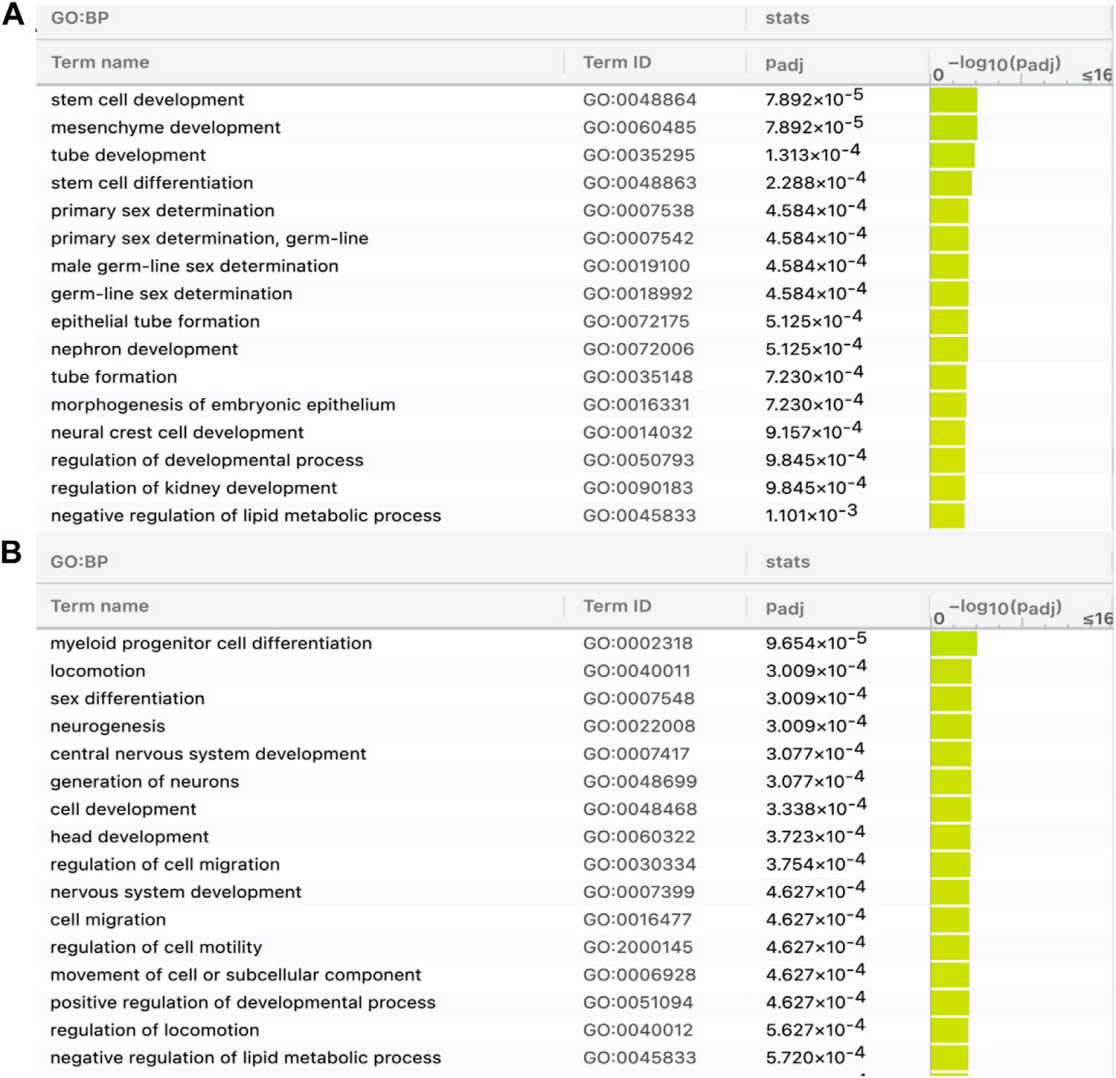
GO analysis of genes containing DBSs for the 376 lncRNAs shared by modern horses, modern donkeys, and Przewalski horses. **(A)** The enriched GO terms of genes in horses with DBSs that have hits (counterparts) in modern donkeys and Przewalski horses. **(B)** The enriched GO terms of genes in horses with DBSs that do not have hits (counterparts) in modern donkeys and Przewalski horses (see Table 4).

The 376 lncRNAs (shared in modern horses, modern donkeys, and Przewalski horses) have more DBSs in the 83 positively selected or NCDS genes in horses than in donkeys and Przewalski horses and many of the DBSs in horses are absent in donkeys and Przewalski horses (Table 4); this prompted us to examine whether the target genes with DBSs in the three species and the target genes with DBSs only in the modern horses are enriched in different functions (Supplementary Tables S9, S10). The results show that the target genes with DBSs in the three species are enriched in GO terms such as “stem cell development” and “tube development,” and “primary sex determination,” and the target genes with DBSs specifically in the modern horses are enriched in GO terms such as “locomotion,” “neurogenesis,” “central nervous system development,” and “generation of neurons” (Figure 6). These GO terms reflect the unique traits of domesticated horses. The results suggest that domesticated horses have more evolved nervous system and running ability compared with donkeys and the undomesticated Przewalski horses, which is consistent with the consensus that domestication changes animal cognitive and behavioral characteristics.

## 4 Discussion

Since a short domestication period, with very limited change in the genome, can make an animal (e.g., dogs, cats, horses) produce many breeds with extremely different phenotypes, it is of great interest to uncover the gene expression regulation mechanisms involved in domestication. The process of animal domestication probably goes through two stages (Zeder, 2015; Pendleton et al., 2018). The first stage is the process of habituation to humans, which produces fear of humans and a reduction in reactive aggression against humans, thereby improving docility. This stage may involve physiological changes that are eventually fixed genetically. The second stage is a longer “breed formation” stage. The two stages are thought to involve distinct genetic changes (Fitak et al., 2020).

The domestication of different animals has commonalities and differences. Some researchers have proposed the NCDS genes as a group of genes reflecting domestication commonalities (Wilkins et al., 2014). However, the neural crest cell hypothesis is not without debate (Johnsson et al., 2021; Wilkins et al., 2021). In addition, exactly what changes these genes undergo in their gene expression regulation remains unclear. In the Equus genus, horses and donkeys have been domesticated, but Przewalski horses remain undomesticated. Thus, the genomes of Przewalski horses and ancient horses provide valuable data for examining the potential regulation of the NCDS genes and the neural crest cell hypothesis.

Computational and comparative genomic analyses are an important complement to and provide valuable clues for experimental studies on animal domestication. This study generates novel results suggesting that changes in the epigenetic regulation of domestication-related genes are an important aspect of horse domestication and that domestication probably leads to more and stronger epigenetic regulation. For horses, the regulation changes are enriched in nervous system development-related genes, which should be common during domestication, and exercise-related genes, which reflect human-specific needs for horse domestication. The results and conclusions from horse domestication may likely apply to the domestication of other animals.

It is worth noting that this study only analyzed high-affinity DBSs of lncRNAs. On the one hand, this made the analysis results quite reliable, but on the other hand, many DBSs with low affinity may be missed, which may also be associated with domestication. LncRNAs and target genes with low-affinity DBSs may reflect more recent, weaker epigenetic regulation. More in-depth analyses of domestication mechanisms can be performed with the identification of more and better ancient DNA sequences of horses and other animals. The methods of this study can be used to analyze the domestication of other animals.

## Data Availability

The original contributions presented in the study are included in the article/Supplementary Material, further inquiries can be directed to the corresponding author.
